# The novel EHEC gene *asa* overlaps the TEGT transporter gene in antisense and is regulated by NaCl and growth phase

**DOI:** 10.1038/s41598-018-35756-y

**Published:** 2018-12-14

**Authors:** Sonja Vanderhaeghen, Barbara Zehentner, Siegfried Scherer, Klaus Neuhaus, Zachary Ardern

**Affiliations:** 1 Lehrstuhl für Mikrobielle Ökologie, Wissenschaftszentrum Weihenstephan, Technische Universität München, Weihenstephaner Berg 3, 85354 Freising, Germany; 2 ZIEL – Institute for Food & Health, Technische Universität München, Freising, Germany; 3 Core Facility Microbiome/NGS, ZIEL – Institute for Food & Health, Technische Universität München, Weihenstephaner Berg 3, 85354 Freising, Germany

## Abstract

Only a few overlapping gene pairs are known in the best-analyzed bacterial model organism *Escherichia coli*. Automatic annotation programs usually annotate only one out of six reading frames at a locus, allowing only small overlaps between protein-coding sequences. However, both RNAseq and RIBOseq show signals corresponding to non-trivially overlapping reading frames in antisense to annotated genes, which may constitute protein-coding genes. The transcription and translation of the novel 264 nt gene *asa*, which overlaps in antisense to a putative TEGT (Testis-Enhanced Gene Transfer) transporter gene is detected in pathogenic *E. coli*, but not in two apathogenic *E. coli* strains. The gene in *E. coli* O157:H7 (EHEC) was further analyzed. An overexpression phenotype was identified in two stress conditions, i.e. excess in salt or arginine. For this, EHEC overexpressing *asa* was grown competitively against EHEC with a translationally arrested *asa* mutant gene. RT-qPCR revealed conditional expression dependent on growth phase, sodium chloride, and arginine. Two potential promoters were computationally identified and experimentally verified by reporter gene expression and determination of the transcription start site. The protein Asa was verified by Western blot. Close homologues of *asa* have not been found in protein databases, but bioinformatic analyses showed that it may be membrane associated, having a largely disordered structure.

## Introduction

It is still a challenge to annotate the steadily increasing number of fully sequenced bacterial genomes and to decide whether an open reading frame is a protein-coding gene or not. Automatic annotation programs such as GLIMMER identify putative genes based on characteristics of sequences that are similar to real genes^1^. The information originates from known genes stored in databases like UniProt or NCBI GenBank, which help in completing the picture of the coding space of bacterial genomes. In practice, misannotations can occur by falsely annotating untranslated gene-like sequences or by overlooking true genes with unusual sequence compositions.

Many efforts have been made to identify and characterize unknown genes and the proteins they encode. For instance, Deutschbauer *et al*.^2^ tested the fitness of genomic knockout mutants on different growth substrates, alternative electron acceptors, stressors and conditions that affect motility. Another approach comes from Hücker *et al*.^3^ who identified constitutively and differentially expressed novel proteins by RNAseq and RIBOseq analysis after growth under various conditions. The genes were further validated by BLAST searches, machine learning, protein feature and regulatory element predictions, and calculations of evolutionary conservation. The combination of experimental and *in silico* methods complement each other to reveal information on the features and cellular functions of previously unknown genes.

Bacterial genes are densely packed in the genome. Typically, more than 90% of a prokaryotic sequence is covered with gene sequences reading in at least one direction. The novel genes discovered in the studies cited above however are all intergenic with respect to annotated genes. Automated annotation of bacterial genomes usually prohibits non-trivial overlapping gene pairs (overlap ≥ 90 bp)^4^. The program decides for one of the open reading frames, for instance, the more conserved one^1^. It was long thought that overlapping gene pairs are unlikely in bacteria with evolutionary constraint leading to a reduced rate of nonsynonymous mutations^5^, a lower fixation rate of mutations^6^ and a diminished optimality of adaptation processes^7^. In recent years however, a few bacterial overlapping genes have been discovered, often as a bycatch in other experiments^8^.

The model organism we use is the human pathogen *Escherichia coli* O157:H7 str. EDL933 (EHEC), which causes a serious form of hemorrhagic gastroenteritis. About 10–15% of infected people develop hemolytic uremic syndrome (HUS) which can lead to kidney failure and even death^9^. The pathogen is transmitted by food, dairy products, animal contacts, contaminated water, via the environment or from person to person^10^. Young children are particularly affected: In 2016, the Robert Koch Institute reported 1816 EHEC infections for Germany, 29% were children younger than five years old^11^.

*E. coli* EDL933 has a chromosome length of about 5 Mbp, a 92 kbp plasmid and a total of 5772 annotated genes^12^. In contrast, only nine overlapping gene pairs have been described in the literature, in all known strains of *E. coli*^13–22^. These genes are often short, not essential and weakly expressed, which makes them difficult to detect^19,22^.

Here we present evidence for the novel gene *asa* (Arginine and Sodium chloride Associated gene) which is completely embedded in the −2 frame of a putative TEGT (Testis-Enhanced Gene Transfer) transporter-encoding gene (frame +1, the ‘mother gene’) in *E. coli* O157:H7 strain EDL933.

## Results

### Transcriptomics (RNAseq) and translatomics (RIBOseq) reveal the presence of a short overlapping gene

**Figure 1:**
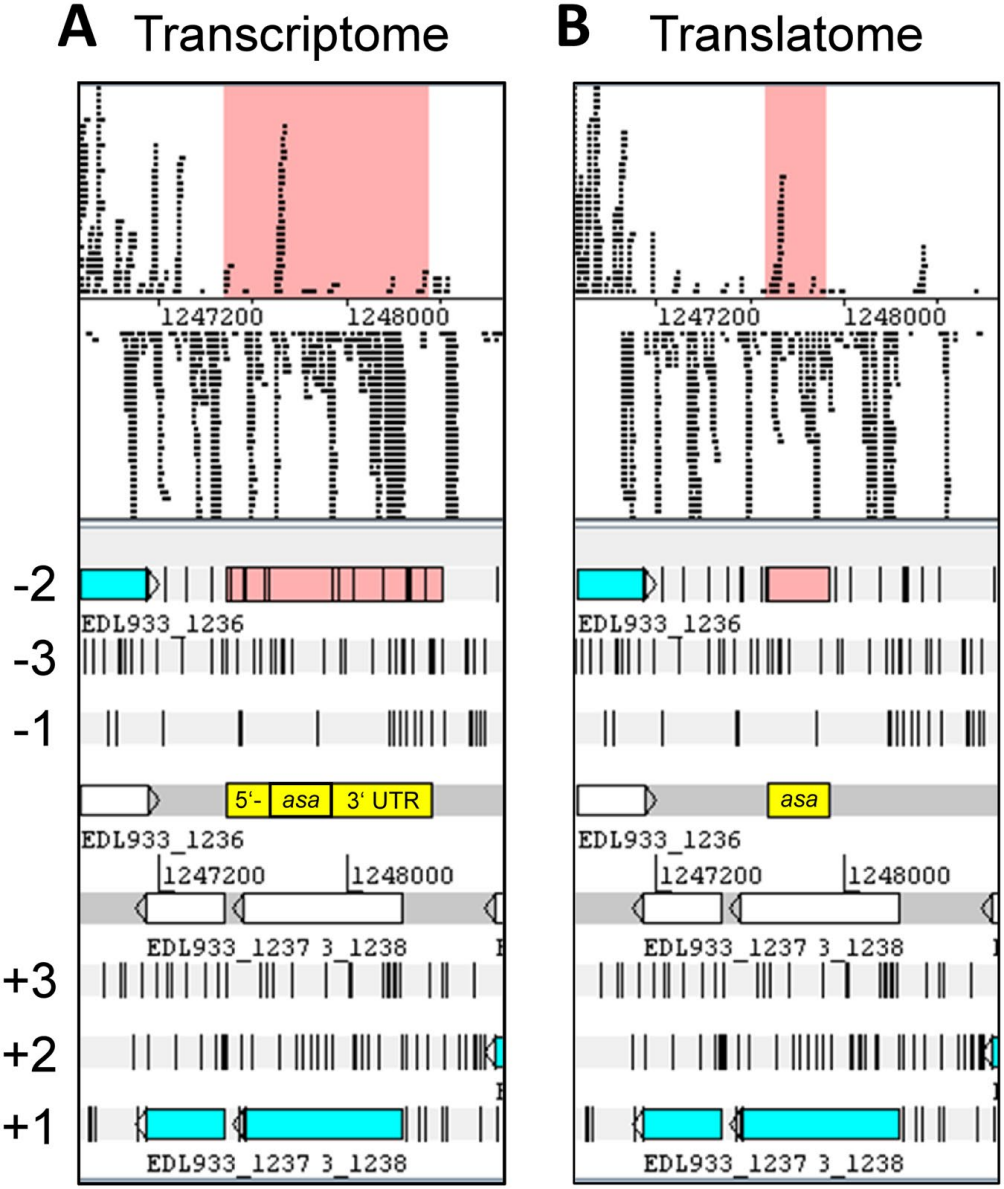
Expression signals of *asa* in EHEC. RNAseq (**A**) and RIBOseq (**B**) signals of *asa* in *E. coli* O157:H7 str. EDL933. Cells were harvested at the transition from the late exponential to early stationary phase. The reads are visualized using bam files in Artemis version 17.0^70^. The putative overlapping gene *asa* is highlighted in salmon or yellow, with its 5′- and 3′-UTRs in case of the transcriptome.

The novel gene *asa* overlapping in antisense to an annotated gene was discovered by strand specific RNAseq and RIBOseq in EHEC. The gene has a clear signal for transcription (RPKM value 14.63) and translation (RPKM value 13.58; Fig. 1). The RPKM value is the number of reads of a given ORF normalized to gene-length and total reads in RNAseq or RIBOseq experiments, i.e. reads per 1 kbp gene length per million reads sequenced.

To validate gene expression, RNAseq and RIBOseq datasets of four further *E. coli* strains (O157:H7 str. Sakai, LF82, K12 substrain MG1655, K12 substrain MC4100) were compared to our model strain. *E. coli* strain Sakai and LF82 are also gastrointestinal pathogens^23,24^, but MG1655 and MC4100 are apathogenic K12 derivatives. *E. coli* O157:H7 str. Sakai is of the pathotype EHEC and isolated in an outbreak derived by contaminated sprouts. *E. coli* LF82 is adherent/invasive (AIEC) and was isolated from patients with Crohn’s disease^25^. The closely related species *S. enterica* strain 14028 S has a truncated *asa* homologue (Supplementary Fig. S1) and was used as negative control. The homologues of *asa* in the pathogenic *E. coli* strains Sakai and LF82 showed a signal for translation (RPKM 8.40 and 18.84). There was no evidence for transcription or translation in the RNAseq or RIBOseq data of the apathogenic *E. coli* strains MG1655 and MC4100 or in *S. enterica* (Supplementary Fig. S2).

### Genome organization of *asa* in *E. coli* and *S. enterica*

**Figure 2:**
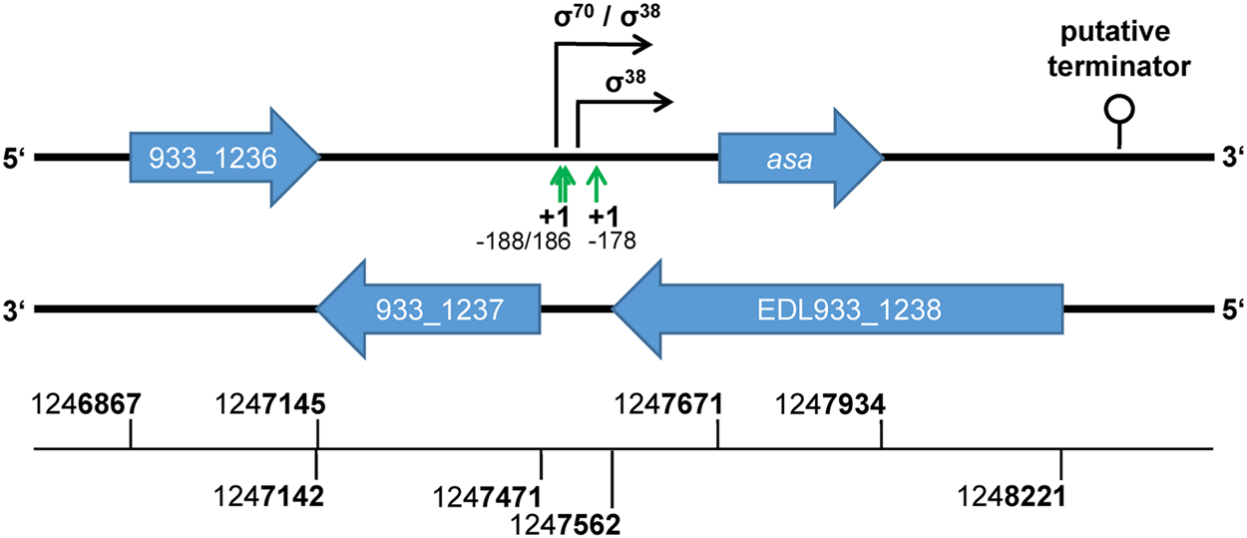
Organization of the genetic region around the overlapping gene pair *asa*/EDL933_1238. Three putative transcriptional start sites (+1 sites) of *asa* were determined 178, 186 and 188 bp upstream of the start codon of *asa* (green arrows). One σ^70^ and two σ^38^ promoters (black arrows) were identified in this region. A putative terminator (loop) was predicted 430 bp downstream of *asa*. Downstream of EDL933_1238 is a gene for tRNA 2-thiouridine synthesizing protein E (EDL933_1237), which has a trivial overlap with a gene for a putative acyl-phosphate phosphohydrolase (EDL933_1236) in antisense. Upstream of EDL933_1238 is a phage integrase (EDL933_1239, not shown) belonging to the O-island #44. Numbers at the bottom of the figure refer to the nucleotide positions of the genes in the *E. coli* str. EDL933 genome.

The gene *asa* (start/stop position 1247671/1247934, given for EDL933) is completely embedded in antisense to a putative carrier/transport protein belonging to the TEGT family with locus tag EDL933_1238 (the ‘mother gene’, Fig. 2). The mother gene is annotated as *yccA* in the EDL933 genome published previously by Perna *et al*.^26^ and it was predicted to encode a transmembrane protein. The Eukaryotic homolog is a Ca^2+^ channel, regulating the cellular Ca^2+^ homeostasis^27^ and is a negative regulator of apoptosis as a BAX inhibitor in eukaryotes and in *E. coli*^28,29^.

The reading frame of *asa* is −2 relative to the +1 frame of its mother gene *yccA*, i.e. the first codon position in *yccA* corresponds to the second position in the overlapping antisense codon of *asa*. There is a tRNA 2-thiouridine synthesizing protein E gene (EDL933_1237) downstream of EDL933_1238, which trivially overlaps a putative acyl-phosphate phosphohydrolase gene (EDL933_1236) in antisense (Fig. 2). Upstream of the mother gene is the O-island #44 encoding the prophage CP-EDL933M.

The synteny of the *asa* region of all *E. coli* strains previously mentioned and the *S. enterica* strain was compared (Supplementary Fig. S3). The gene pair, as well as the region downstream of the mother gene containing the acyl-phosphate phosphohydrolase and the sulfotransferase are always conserved, but the homolog of *asa* is truncated in *Salmonella*. The upstream region of EDL933 is only shared with its closely related strain Sakai. The K12 strains and strain LF82 have genes encoding a serine tRNA and a hydrolase-1 operon in this position. The hydrolase operon, in which the prophage integrated in EDL933 and in Sakai, is partly present in both EHEC strains^26,30^. *S. enterica* has a hypothetical protein, the serine tRNA and a pathogenicity island upstream of the mother gene, i.e., the gene in which the *asa* homolog is embedded.

### Overexpression phenotypes in NaCl and in L-arginine

**Figure 3:**
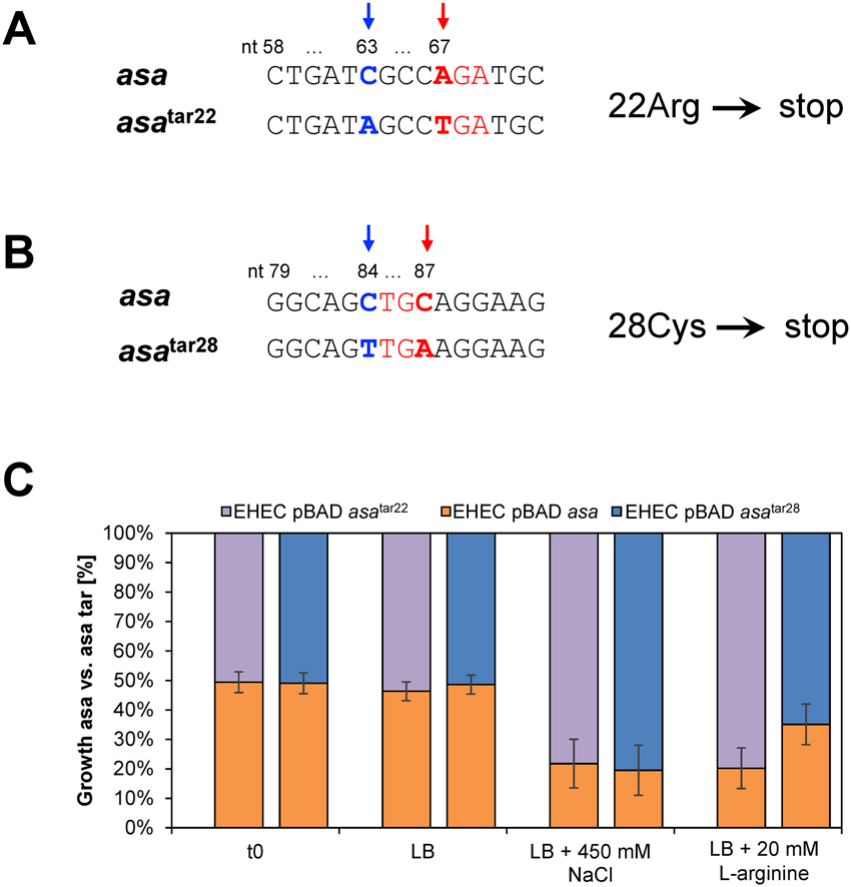
Competitive growth experiments of *asa* wild type against translationally arrested mutants (*asa*^tar^) in the absence and presence of NaCl and L-arginine. (**A**) The nucleotide changed at position 67 (A → T, bold red) introduces a stop codon leading to translational arrest at amino acid 22 (*asa*^tar22^). At nt position 63, a neutral C → A mutation was introduced (bold blue) to determine differences in competitive growth after sequencing. (**B**) The nucleotide changed at position 87 to introduce a stop codon (C → A, bold red) leading to translational arrest at amino acid 28 (asa^tar28^). At nt position 84, a neutral C → T mutation (bold blue) was introduced to analyze competitive growth. (**C**) Competitive growth after overexpression causes phenotypes of *asa* versus *asa*^tar^ mutants shown as ratio of wild type:mutant [%] in the absence and in presence of stressors. EHEC with pBAD-*myc*/His C-*asa* (orange) was grown competitively (initial ratio t_0_ = 1:1) against the translationally arrested mutant pBAD-*myc*/His C-*asa*^*tar22*^ (violet). In a second experiment EHEC with pBAD-*myc*/His C-*asa* was grown competitively against pBAD-*myc*/His C-*asa*^*tar28*^ (blue). Gene expression was induced with 0.002% arabinose and the culture was harvested after 22 h. The final ratio of wild type and mutant was determined as relative peak height obtained by Sanger sequencing of the mutated nucleotides. Shown are the mean values of the wild type:mutant ratios and standard deviations of biological triplicates.

Overexpression phenotypes of *asa* in the presence of various stressors (17 in total) were determined by competitive growth of EHEC pBAD-*myc*/His C with wild-type *asa* as insert against those with a mutated *asa* insert (i.e., causing a premature translational arrest, *asa*^tar^). Competitive growth experiments have the advantage of being very sensitive in detecting a phenotype in comparison to single strain growth experiments. The same cellular environment - a vector with an insert of the same length, which only differs by two point mutations (i.e. *asa*^tar22^) from the wild type gene, excludes influences on bacterial fitness caused by the plasmid (Fig. 3A). Positional effects of the translational arrest were excluded by creating a second translationally arrested mutant with a stop codon at an alternative position (*asa*^tar28^, Fig. 3B).

The competitive assay revealed a fitness disadvantage of EHEC with overexpressed *asa* grown in LB + 450 mM NaCl and in LB + 20 mM L-arginine (Fig. 3C). When stressed with NaCl, the ratio *asa*:*asa*^tar22^ was about 1:4 and with L-arginine as stressor about 1:1.9, respectively. For the alternative stop codon mutation *asa*^tar28^, the ratio was about 1:4 in NaCl and 1:4 in L-arginine, respectively. Both phenotypes were only observed in NaCl and L-arginine. Wild type and mutant remained at the ratio 1:1 in plain LB medium (Fig. 3C) or in the presence of further stressors (preliminary experiments, data not shown).

### *asa* expression may be regulated by three putative promoters

**Figure 4:**
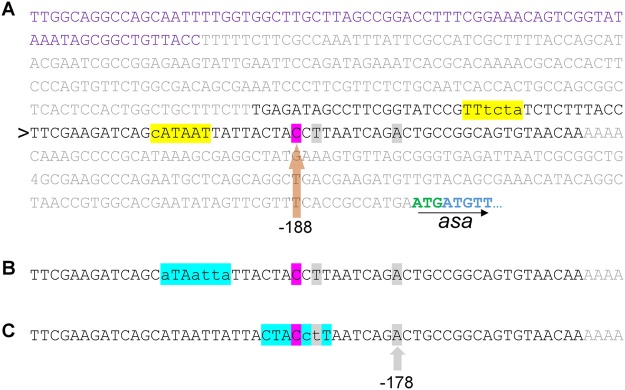
Promoter region of *asa*. (**A**) The region containing the putative promoter (black letters, 92 bp) and the terminator region of EDL933_1236 (violet letters, used as negative control) were cloned into pProbe-NT upstream of a GFP gene. The line marked by the arrowhead is copied in (**B**,**C**) to show additional details. The promoter activity measured originates probably from one of three putative promoters (compare to Fig. 2): A σ^70^ promoter (highlighted in yellow in **A**) or either one of two σ^38^ promoters (highlighted in blue **B**,**C**). Capital letters in each of the highlighted promoter sequences match to the respective consensus sequences TATAAT/TTGAAT for σ^70^ or CTACACT for σ^38^. The +1 site determined with Cappable seq is highlighted in violet; those determined with 5′RACE are highlighted in grey.

There are three putative transcriptional start sites. The one determined with Cappable seq in LB was 188 bp upstream of the start codon (Fig. 4A). The +1 site determined by 5′RACE in EHEC grown in LB was located 186 bp upstream, and the +1 site determined using cells grown in LB +450 mM NaCl was located 178 bp upstream of the start codon. None of the putative transcriptional start sites are located antisense in the ORF of the mother gene of *asa* (compare Fig. 2). The 5′-UTR does not show obvious evidence for a second open reading frame.

Three putative promoter sequences were discovered: A σ^70^ promoter predicted by BProm (LDF = 2.10) is localized 9 bp upstream of the +1 site determined by Cappable seq, which is 188 bp upstream of the start codon (Fig. 4A). A putative σ^38^ promoter was found 7 bp upstream of the same +1 site (Fig. 4B). Lee and Gralla^31^ identified the consensus sequence of σ^38^ dependent promoters in an electrophoretic mobility shift assay. The sequence ATAATTA found here has an average conservation of only 36% in an alignment of 41 promoters. A potential second σ^38^ promoter was found 7 bp upstream of the +1 site determined with 5′RACE in the presence of 450 mM NaCl, which is 178 bp upstream of the start codon (Fig. 4C). According to Lee and Gralla^31^, the nucleotide sequence CTACCTT of this putative promoter had an average conservation of 59%.

The terminator sequence next to *asa* was 430 bp downstream of the stop codon with a predicted free energy of ∆G° = −19.7 (Fig. 2). The region between *asa* and the terminator sequence was checked for further open reading frames, but there were no obvious ones. Thus, it is questionable whether this terminator is responsible for transcriptional cessation of the *asa* mRNA.

The promoter region of *asa* is conserved in all five *E. coli* strains (Supplementary Fig. S4). The nucleotides forming the different transcriptional start sites in strain EDL933 are conserved in the other *E. coli* strains, as well as the σ^70^ promoter and the second σ^38^ promoter of the 178 bp upstream position. The first σ^38^ promoter of the position 186/188 bp upstream is conserved in all strains except LF82, in which a “C” nucleotide is inserted additionally at position 7 in the promoter region. This renders it improbable that the sequence functions as σ^38^ promoter^31^. In *S. enterica*, the σ^70^ promoter is absent; there are several nucleotide differences compared to EDL933. Both σ^38^ promoter regions in *S. enterica* also have differences in single nucleotides compared to EHEC (“point mutations”) which decreases the probability that those regions function as promoters (Supplementary Fig. S4). However, a low interspecies conservation of promoters has been previously observed^32^.

### Gene regulation of *asa* depends on growth phase and growth medium

**Figure 5:**
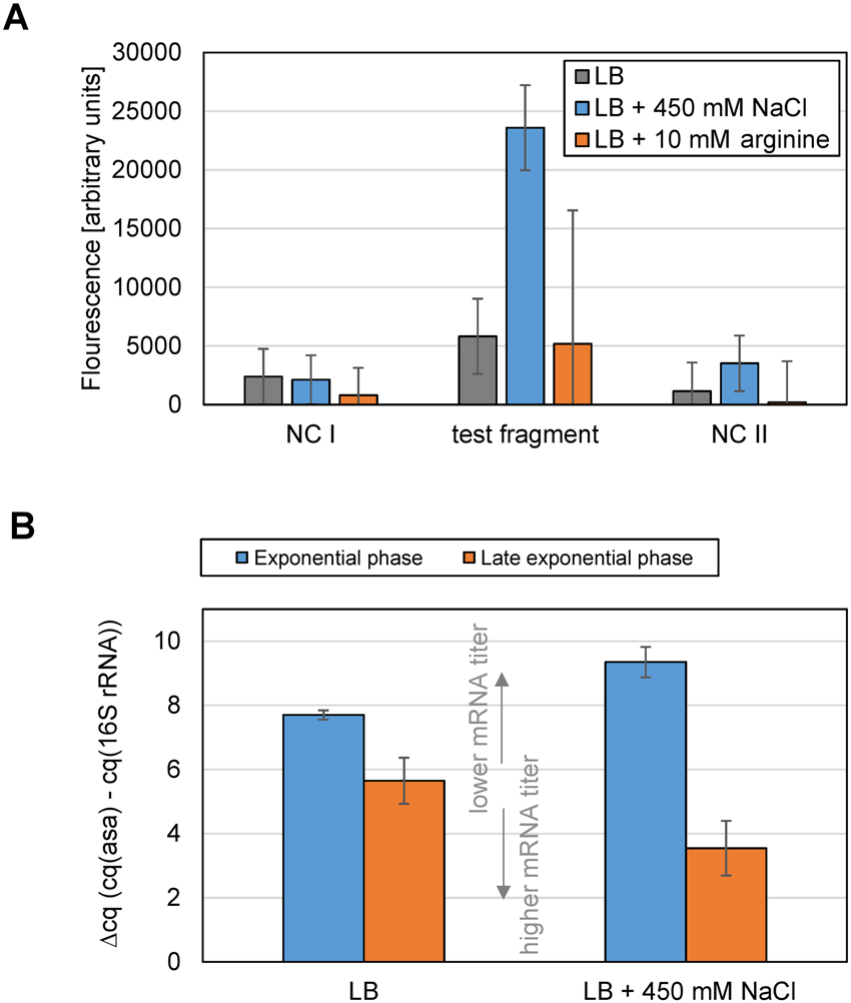
Regulated transcription of *asa*. (**A**) Promoter activity of the cloned putative promoter region (black letters in Fig. 4A) was measured as fluorescence intensity of *E. coli* Top-10 + pProbe-NT::*asa*^P^ (OD_600 _= 0.8) after growth in LB (grey), in LB + 450 mM NaCl (blue) or in LB + 10 mM L-arginine (orange); NC I: negative control using the terminator region of the gene EDL933_1236 (violet letters in Fig. 4A), NC II: pProbe-NT without insert. The mean values and standard deviations of all three replicates are shown here. (**B**) RT-qPCR threshold cycles of *asa* normalized to the 16S rRNA gene (∆cq). Lower ∆cq values indicate higher amounts of mRNA and *vice versa*. EHEC was grown in LB medium (left columns) or LB + 450 mM NaCl (right columns). Approximately 10^8^ cells were harvested for total RNA extraction either at exponential phase (OD_600_ = 0.2–0.3, blue) or at late exponential phase (OD_600_ = 0.7 – 0.8, orange). The RNA was reverse transcribed into cDNA and quantified using RT-qPCR. The mean values and standard deviations of all three replicates are shown here.

The sequence containing the putative promoter sites was cloned in a pProbe-NT vector upstream of a promoter-less green fluorescent protein (GFP)^33^. In this vector, the promoter activity is proportional to the GFP fluorescence measured. Both negative controls, either a vector with a terminator site inserted (NC I) or empty vector (NC II), showed very low background GFP expression. The conditions tested for the *asa* promoter were either salt or arginine supplemented LB, and they were compared to plain LB as control. The fragment of 92 bp in length and starting 160 bp upstream of the *asa* start codon (Fig. 4A) has a weak promoter activity in plain LB-medium and in L-arginine supplemented LB (Fig. 5A). However, the activity increased about 4-fold in NaCl in comparison to plain LB.

Due to the strong phenotype in the presence of NaCl, this condition was selected to be compared by RT-qPCR to plain LB. We also tested whether the regulation of transcription is growth phase dependent in either plain LB or medium supplemented with salt. The gene *asa* is upregulated in later exponential phase compared to the exponential phase for both conditions, i.e. plain LB (2-fold) and LB with salt (6-fold, Fig. 5B, Supplementary Table S1). The mRNA titer in plain LB is higher when compared to LB supplemented with NaCl during exponential phase (1.6-fold more in LB). In contrast, for the late exponential phase, 2.1-fold more transcript was detected in LB with salt compared with LB alone. Two negative controls were used in this experiment: the qPCR of each 16S rRNA sample which was not reverse transcribed and a 59 bp region which has no RNAseq signal when harvested at late exponential phase after growth in LB; both remained negative (Supplementary Table S1).

### Overexpression of Asa results in two products

**Figure 6:**
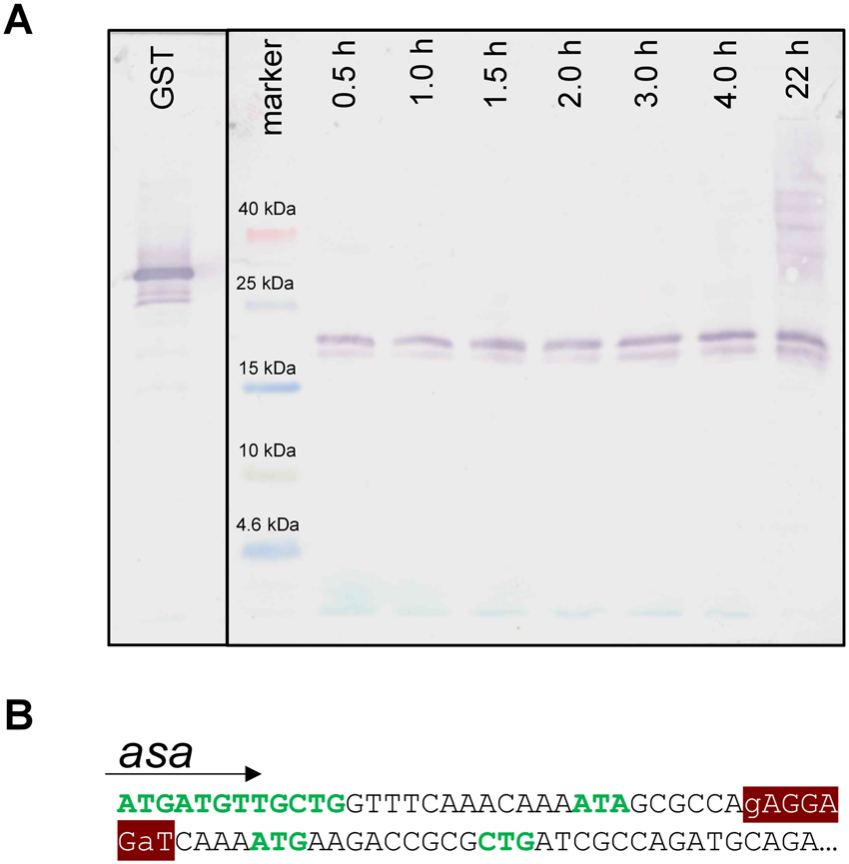
Western blot and alternative start codons of Asa. (**A**) Asa was linked to a C-terminal SPA tag, overexpressed, detected with an anti-FLAG antibody, and visualized with NBT/BCIP. Aliquots were taken 0.5 h, 1 h, 1.5 h, 2 h, 3 h and 4 h after induction with 0.002% arabinose (w/v). To avoid overexposure of the positive control, the blot was cut (black line) and both pieces were developed separately until bands appeared. Both parts were aligned and photographed together and the complete blot is shown. From left to right: GST, positive control (30 kDa); marker, Spectra Low Range Marker (Thermo Fisher Scientific), Asa::SPA, time points taken as indicated after induction. The aliquot volume was adjusted to time point 0.5 h based on OD_600_. (**B**) The first 75 bp of *asa*. Possible start codons are shown in green letters. The Shine-Dalgarno sequence (∆G° = −5 kcal/mol) is highlighted in dark red. Small letters in that region show mismatches to the consensus (TAGGAGGT).

The *asa* encoded protein was linked to a C-terminal SPA tag (Sequential Peptide Affinity) which consists of a calmodulin binding peptide, a TEV protease cleavage site and three modified FLAG sequences^34^. Overexpression of the 17.5 kDa fusion protein (10 kDa Asa +7.5 kDa SPA) was detected in a Western blot using an antibody against the tag. Asa::SPA fusions were detected at different time points. Two bands were stained on the blot (Fig. 6A). The upper and more prominent band migrated at approximately 20.6 kDa and the lower band at 17.8 kDa. We assume that the upper band should reflect the full-length product. We raise the possibility that the smaller product may be due to an alternative start codon, located further downstream. Indeed, a strong Shine-Dalgarno sequence (AGAGGAGAT, ∆G° = −5 kcal/mol) was found 5 bp upstream of another ATG codon. This alternative start codon lies 45 bp downstream of the first start codon, which reflects the longest possible open reading frame (Fig. 6B). There is no predicted Shine-Dalgarno sequence upstream of the first start codon. The protein product of the alternative start codon would be, theoretically, 1.65 kDa smaller. Which start codon is naturally preferred in the cell is unknown, but the Western blot indicates that both are able to be expressed.

### Asa is predicted to encode a secreted, disordered protein with one transmembrane region and one disulfide bridge

**Figure 7:**
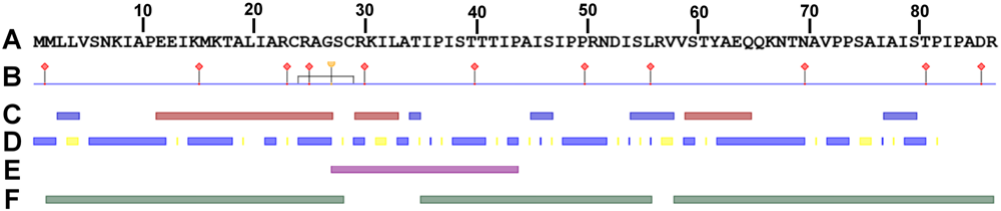
Protein features of *asa* analyzed with PredictProtein^73^. (**A**) Amino acid sequence (**B**) Binding sites; ruler, AA numbering; needle with red square, protein binding site; needle with yellow circle, DNA binding sites; grey bridge, disulfide bond. (**C**) Secondary structures; blue, β-strand; red, α-helix. (**D**) Solvent accessibility; blue, exposed regions; yellow, buried regions. (**E**) Transmembrane regions (violet). (**F**) Disordered regions (green).

Searches using blastp did not reveal any close homologs of the protein putatively encoded by *asa*. Further, there are no hits to Pfam or conserved domains. To get more information about Asa, protein features were predicted using PredictProtein (Fig. 7). Accordingly, Asa is predicted to be predominantly disordered (91% amino acids in disordered regions). There is one α-helix from amino acid 11 to 26 (possibly absent in the short form), one disulfide bond bridging amino acid 23 and 29 (thus, present in the short form) and one predicted transmembrane region from amino acid 26 to 42 (also present in the short form). Interestingly, *asa* is predicted to encode a secreted protein. Hints for interactions are given by the prediction of DNA and protein binding sites within this protein. Asa has one DNA and eleven putative protein binding sites of various sequence motifs predicted.

## Discussion

The short gene *asa* was identified by RNAseq and RIBOseq in EHEC. Many RNAseq and RIBOseq signals observed in experiments cannot be assigned to known coding regions and these features have been described as ‘pervasive’^35,36^. However, this previously thought to be ‘non-coding’ RNA has been increasingly assigned a function^37,38^ and such transcription products may also encode peptides^39^. There are two major lines of experimental evidence which suggest functionality of an expressed gene: The presence of a phenotype, i.e., contribution to a cellular process, and specific regulation (e.g., under the circumstances in which a phenotype was observed). Unregulated genes would confer an energy cost and be selected against if the expressed element did not have a positive function for the cell^40^.

Evidence for involvement in a cellular process can be either a phenotype produced by a deletion which decreases gene dosage or by an overexpression which increases gene dosage^41^. Here, we found a strong fitness disadvantage of overexpressed *asa* in both salt stress and the presence of L-arginine. This seems to contradict the increased promoter activity in sodium chloride. However, the amount of *asa* product present after artificial overexpression surely exceeds natural levels, which, in turn, decreases the fitness. This becomes evident when comparing Western blot results with the translational level found by RIBOseq (see below). Consequently, the translationally arrested mutants can overgrow the wild type in competitive assays. Salt and increased levels of arginine were found to cause a phenotype after overexpression. Similar environmental cues might also be important when *E. coli* enters the intestinal tract. This would lead to osmotic stress and an increase of nutrient concentrations, necessitating internal changes in the gene expression profiles^42,43^.

Gene regulation of *asa* depending on growth phase and conditions was shown by RT-qPCR. There was an up-regulation from exponential to late exponential phase in LB and in LB supplemented with sodium chloride. During the exponential phase, there is a lower *asa* mRNA titer under salt stress in comparison to LB. The promoter activity tests and RT-qPCR show a high mRNA titer in salt stress which exceeds the titer in LB during late exponential phase.

Growth on L-arginine also resulted in a negative fitness effect when *asa* was overexpressed. However, the promoter activity is comparable to the activity in LB medium. The phenotype was measured after 22 h growth. There is a possibility that the low promoter activity during exponential phase in arginine-medium is higher in growth phases not tested or that there is even another promoter outside the cloned region which may be active in the presence of arginine.

Despite using two independent methods - Cappable seq and 5′ RACE - an exact nucleotide of the +1 site could not be determined. All three biological replicates of the Cappable seq had a peak 188 bp upstream of the start codon (data not shown) which was very close to the nucleotide (186 bp) determined with 5′RACE of cells grown in LB medium. Ettwiller, Buswell *et al*.^44^ estimated the precision of Cappable seq in a range of five nucleotides around the +1 site. They further hypothesized that a promoter can have more than one transcriptional start site and found that for 60% of the promoters the RNA polymerase does not start transcription at a specific single nucleotide, which corresponds to our findings.

There are two putative promoters that have the potential to initiate the gene expression of *asa* - a σ^70^ promoter and a σ^38^ promoter. The σ^70^ promoter is the promoter used for most genes in *E. coli* (consensus sequence at −10/−35 site: TATAAT/TTGAAT^45^). The σ^38^ promoter regulates general stress responses including salt stress (consensus sequence at −10 site: CTACACT^31^, without any conserved −35 site). A σ^38^ promoter can be induced under stress conditions as an alternative to the σ^70^ promoter used in non-stress growth^46^. Both promotors are very similar and may change from one into the other due to slight differences in the consensus sequence^47^. There is a +1 site 178 bp upstream of the start which was identified after growth in salt stress and a related σ^38^ promoter in front of the start site. It is known that genes can be regulated by more than one promoter, such as in sporulation-associated genes in *Bacillus subtilis*^48^. There is therefore a possibility that *asa* switches its promoter dependent on the environmental conditions.

Interestingly, signals for translation of *asa* were found in the pathogenic strains EDL933, Sakai and LF82, but no signals appeared in apathogenic *E. coli* K12 MG1655 and MC4100 (Supplementary Fig. S2). The amino acid sequence of the *asa* encoding open reading frame (Supplementary Fig. S1) and the promoter region (with one exception, see results, Supplementary Fig. S4) are conserved within the *E. coli* strains. The gene region of *asa* itself is shared between EDL933 and Sakai. The strains LF82, MG1655 and MC4100 only share the general region (Supplementary Fig. S3). However, an experimental analysis of three pathogenic and two apathogenic strains is not sufficient for a generalization about *asa*’s involvement in virulence. Further, there are no known pathogenicity-related genes nearby *asa* or homologues in Sakai and LF82 which would have supported this type of suggestion.

There are various indications that the *asa* gene is protein-coding and not a non-coding RNA (ncRNA). For instance, translation signals of RIBOseq experiments provide strong evidence that *asa* encodes a small protein of 87 amino acids^8,38^. In addition, there is a phenotype after competitive growth of overexpressed *asa* against two different overexpressed but translationally arrested *asa* mutants. Translationally arrested mutants express the full-length mRNA, but produce only a truncated protein. This is clear evidence that the phenotype is caused by the protein and not by a ncRNA, since out of more than 264 nt (comprising the full-length ORF of *asa*), only 2 nt have been changed to obtain arrested mutants. A substantial change in ncRNA function, by such minor changes, tested for two different locations here, is unlikely^49^. Further, Asa was detected by Western blot which is clear evidence for the presence of a protein. Already 0.5 h after induction, there are significant amounts of Asa present and the product is still stable 4 h after induction (Fig. 6). However, our RIBOseq results (Fig. 1) show that this protein is naturally present in relatively low concentrations in the cell.

In the Western blot, two bands of SPA-tagged Asa were visible and the size of this small protein did not fully correspond to the expected size, which needs explanation. Interestingly, isoforms for small proteins have been reported recently for bacteria. SPA-tagged MntS of *E. coli* also showed two bands and the authors suggested either two distinct conformations or modifications^50^. In contrast, for VirF of *Shigella* two isoforms differing in length have been described^51^. In addition to such isoforms, deviations in expected and real migration patterns have been observed multiple times for gel electrophoresis. Apparent molecular weight differences for up to 10% are often detected^52–55^.

Distinctive patterns in the amino acid sequence can give hints of secondary structures or functional elements, exploited by prediction pipelines like PredictProtein. The putative *asa* encoded protein has long disordered regions and one α-helix (at least in the full-length version of Asa) and, therefore, may be predominantly unstructured. Based on the Key-lock theory^56^, it was thought for a long time that proteins must be structured to be functional. The discovery of disordered proteins contradicts this dogma and today it is known that such proteins are involved in a multitude of cellular functions^57^. Interestingly, the predicted features of *asa*, especially the extent of disordered regions, corresponds to some features of the “dark proteome”, as defined by Perdigão *et al*.^58^. A dark protein is a singular protein (i.e., only present in the Swiss-prot database for that study) without any matches to known proteins, structures or structural motifs in further databases. Interestingly, bacteria have a higher fraction of disordered proteins compared to other organisms and proteins with the highest degree of “darkness” (i.e., lowest darkness score) have the highest fraction of transmembrane proteins^58^. Secretion and the presence of transmembrane regions or disulfide bridges are common features of proteins that are unknown and hard to detect^3,58,59^, both of which have been predicted for Asa. One must consider that PredictProtein, like all other programs predicting protein structure *ab initio*, is based on ‘known’ protein features and reliable information about such ‘unknowns’ - without any homologues in databases - cannot be gained solely by computation. Therefore, the characterization of such proteins harbors the potential to discover further so far unknown structural and functional features.

## Materials and Methods

### List of bacteria and incubation conditions

The following bacterial strains were used: *E. coli* O157:H7 strain EDL933 (Collection de l’Institute Pasteur: CIP 106327, GenBank accession number CP008957.1), *E. coli* O157:H7 strain Sakai (Weihenstephan strain collection WS 4518, GenBank accession number NC_002695.1), *E. coli* LF82 (kindly provided by R. Balfour Sartor, GenBank accession number NC_011993.1), *E. coli* TOP-10 (Invitrogen, Paisley, UK). All bacteria were grown in Luria Broth medium (LB) at 37 °C.

### Isolation of genomic DNA and cloning of *asa* in pBAD-*myc*/His C

Overnight cultures of *E. coli* O157:H7 strain EDL933 were mechanically lysed by bead beating with 100 µl of 0.1 mm zirconia beads (Carl Roth, Germany) in a FastPrep-24 Instrument (MP Biomedicals). The genomic DNA was isolated with CTAB (Sigma Aldrich) and subsequent phenol-chloroform extraction using Roti®Phenol (Carl Roth, Germany) and Roti®Phenol plus chloroform/isoamylalcohol (Carl Roth, Germany). The RNA was subsequently removed using RNase A (20 mg/ml, Sigma Aldrich). The gene *asa* was amplified with PCR (primers: 8220+1F-*Nco*I, GAT CCA TGG GGA TGT TGC TGG TTT CAA ACA; 8220+245R-*Hind*III, GCC AAG CTT CTA TCT GTC TGC CGG AAT GG) by adding restriction enzyme cutting sites for *Nco*I and *Hind*III. For all PCRs, the Q5 High-Fidelity DNA Polymerase (New England Biolabs) was used. The vector pBAD-*myc*/His C (Invitrogen) and *asa* were double digested with *Nco*I and *Hind*III (Thermo Fisher Scientific) at 37 °C for 3.5 h and ligated using T4 ligase (Thermo Fisher Scientific). The ligation preparation was desalted by swimming filter dialysis and transformed in *E. coli* TOP-10 by electroporation. The successful cloning was verified by colony PCR (primers: pBAD-C+165F, CAG AAA AGT CCA CAT TGA TT; pBAD-C-R, TGA TTT AAT CTG TAT CAG GC) and Sanger sequencing (Eurofins, Ebersberg).

### Construction of translationally arrested mutants

Mutations leading to a translational arrest were inserted by mutation PCR in two steps modified after Patel *et al*.^60^ and An *et al*.^61^. In a first step, two PCR fragments were synthesized from the plasmid with the wild type *asa* construct. One fragment covered the sequence from the start to the mutation site and a second from the mutation site to the stop of the gene. The mutation primers with a length of 20 nucleotides bind to the gene with mismatches at mutated bases. For both PCR fragments two reverse complement primers which overlap 100% are required. The PCR fragments overlap with the length of the mutation primers and can be merged in a second PCR. For this, the purified PCR fragments of the first step are mixed with all PCR reagents excluding primer. The PCR-like reaction was conducted for 15 cycles allowing overlapping fragments to bind and extend. The resulting products were amplified by adding the flanking primers (8220+1F-*Nco*I, 8220+245R-*Hind*III) and the PCR was continued for a further 20 cycles. The fragment was purified and used for cloning in pBAD-*myc*/His C as described above. To exclude position effects, two translationally arrested (tar) mutant genes were tested which differ by the stop codon position (primer stop codon position 22: 8220KO+52F, CCG CGC TGA TAG CCT GAT GCA; 8220KO+73R, TGC ATC AGG CTA TCA GCG CGG; primer stop codon position 28: 8220alterKO+76F, CAG GCA GTT GAA GGA AGA TAT; 8220alterKO+97R, ATA TCT TCC TTC AAC TGC CTG). Further, two nucleotides were mutated in *asa*^tar22^ as well as in *asa*^tar28^_,_ which lie in the first third of the sequence. One bp was changed to introduce the stop codon, a second bp was changed synonymously to enable correct measurements for competitive growth experiments (see next paragraph).

### Competitive growth assays

In preliminary experiments, 17 stressors were tested to identify a phenotype caused by *asa* (4 mM L-malic acid, 20 mM L-arginine, 20 mM CsCl, 4 mM acetic acid, 4 mM malonic acid, 20 mM 1-methyl imidazole, 500 mM NaCl, 4 mM NaOH, 4 mM Na_3_VO_4_, 0.16 mM sodium salicylate, 0.32 mM perchloric acid, 0.32 mM phytic acid, 100 mM 1,2-propanediol, 20 mM 1-propanol, 20 mM pyridoxine HCl, 0.8 mM ZnCl_2_, and a Staphylococci mixture of *S. aureus* and *S. chromogenes*). Only two stressors, LB plus NaCl or L-arginine (Sigma Aldrich), showed a clear response and were used for competitive growth assays. For this, precultures of EHEC + pBAD-*asa* and EHEC + pBAD-*asa*^tar^ (either *asa*^tar22^ or *asa*^tar28^) were each diluted to an OD_600_ = 1. Cultures of the bacteria with the intact insert and translationally arrested insert were mixed in equal amounts (1:1). An aliquot was frozen and used as time zero control (t_0_). The remaining culture was diluted 1:300 (i.e. 5 µl culture added to 1.5 ml LB medium). One hundred microliters of this dilution was used for inoculation in 10 ml LB + ampicillin (120 µg/ml, Carl Roth) + 0.002% arabinose (w/v, Carl Roth) + one stressor (i.e. 450 mM NaCl instead of 500 mM allowing better growth, or 20 mM L-arginine).

The above cell cultures were incubated at 37 °C, 150 rpm. After 6.5 h incubation, a further 0.002% arabinose (w/v) was added to the cell culture. Twenty-two hours after inoculation, the cells were harvested, plasmids isolated (GenElute^TM^ Plasmid Miniprep Kit, Sigma-Aldrich) and sequenced by Sanger sequencing (Eurofins, Ebersberg). The peak-height ratio of both inserts at the site mutated leading to a translational arrest mutant was used to determine the growth phenotype after 22 h of competitive growth. Each experiment was repeated at least three times.

### Quantitative PCR of reversely transcribed *asa* mRNA

#### Growth conditions for RT-qPCR

The regulation of *asa* was analyzed in LB medium and under NaCl-stress conditions. Due to slow growth in 500 mM NaCl, those overnight cultures were prepared using LB plus 450 mM salt to achieve a pre-adapted culture. Thus, overnight cultures were grown in plain LB medium for two hours, after which 450 mM NaCl was added. The next day, 500 µl of the overnight cultures were transferred into 40 ml fresh LB +450 mM NaCl. Accordingly, EHEC overnight cultures grown in LB were transferred into fresh LB medium and incubated (growth curves can be found in Supplementary Fig. S5). An equivalent cell number present at OD_600_ = 1 in 1 ml culture (≈8·10^8^ cells) was harvested for RNA extraction at either OD_600_ = 0.2–0.3 (exponential phase) or OD_600_ = 0.7–0.8 (late exponential phase). Cell pellets were shock-frozen in liquid nitrogen and stored at −80 °C until RNA isolation.

#### RNA-extraction

For RNA extraction, the cell pellet was resuspended in 1 ml RNAprotect (Qiagen). After vortexing (5 s) and incubation (5 min RT), the mixture was centrifuged for 10 min at 8000 × *g*. The pellet was resuspended in 100 µl TE buffer, supplemented with lysozyme (10 mM Tris/HCl, 1 mM EDTA, pH 8.0; 0.4 mg/ml lysozyme). Subsequent steps were conducted using the SV total RNA Isolation System (Promega) according to the manual. The DNA was digested with TURBO^TM^ DNase (Thermo Fisher Scientific) for 1 h. The RNA concentration was measured with a NanoDrop (Thermo Fisher Scientific). The absence of DNA was checked using a PCR (primers: 8220+1F-*Nco*I, 8220+245R-*Hind*III) with Q5 High Fidelity DNA Polymerase (New England BioLabs), and analyzing the product on a 2% agarose gel. Additionally, RNA samples were tested to be DNA-free by qPCR with 16S rRNA primers (rrHF, AAT GTT GGG TTA AGT CCC GC; rrHR, GGA GGT GAT CCA ACC GCA GG) using an aliquot which was not reverse transcribed.

#### Reverse transcription and quantitative PCR (RT-qPCR)

Regulation of *asa* gene expression was measured by RT-qPCR. A negative control for gene expression was additionally tested: a 59 bp sequence without any RNAseq signal (position 1434446–1435140, cq = 32). The EHEC RNA (1.6 µg) was reverse transcribed with Superscript III (Thermo Fisher Scientific) according to the protocol using a random nonamer primer (50 µM, Sigma Aldrich) in the presence of the Superase In RNase Inhibitor (20 U/µl, Invitrogen). The qPCR reaction mix contained 12.5 µl Sybr^TM^ Select Master Mix (Thermo Fisher Scientific), 0.5 µl of each primer, 2 µl DNA or cDNA and 9.5 µl H_2_O. The qPCR reaction conditions were as follows: Initial denaturation at 95 °C for 5 min, denaturation at 95 °C for 15 s, annealing at 58 °C (negative control) or 61 °C (*asa*) for 30 s, elongation at 72 °C for 30 s using 40 cycles in total, and final elongation at 72 °C for 5 min. A melting curve was acquired for quality control of the final product. The primer efficiency was tested using genomic DNA of EHEC in amounts of 400 ng, 40 ng, 4 ng and 0.4 ng DNA. The primer efficiency of *asa* (qPCR-OLG8220+25F, TAG CGC CAG AGG AGA TCA AA; qPCR-OLG8220+191R, CGT TAG TGT TCT TCT GCT GC), the 16S rRNA gene (rrHF, rrHR, later used for normalization) and the negative control (qPCR-neg8220F, GTC ATC CAC TGC GAC AAG AA; qPCR-neg8220R, GTA CAC TTA GAT TTG ACA ACC GC) was determined as follows: *asa:* 130% (R^2^ = 0.9992), negative control: 99.6% (R^2^ = 0.9993), and 16S rRNA: 87% (R^2^ = 0.9995) at 61 °C and 80% at 58 °C (R^2^ = 0.9981). The standard curves can be found in Supplementary Fig. S6. Gene *asa*, the negative and the normalizer sequence were each amplified in three technical replicates for each qPCR run. Each condition was measured in biological triplicate. The negative control for reverse transcription was a qPCR of all samples without reverse transcriptase (primers: rrHF, rrHR, cq was with one exception, always above 28). The gene regulation was calculated using the ∆cq method^62^.

### RNAseq and RIBOseq

The transcription and translation of *asa* and of homologues in *E. coli* and *Salmonella enterica* strains were detected by RNAseq and RIBOseq experiments. The data of EHEC^63^, *E. coli* O157:H7 strain Sakai^3^ and *E. coli* LF82 (unpublished) are from our lab. RNAseq and ribosome profiling sequence reads in *E. coli* K12 substrain MG1655^64^ and substrain MC4100^65^ and of *S. enterica* 14028S^8^ were downloaded from the sequenced reads archive, SRA, NCBI. All accession numbers are listed in Supplementary Table S2. The bash script for the ribosome profiling (RIBOseq) analyses, with input file instructions and tool version numbers, is available as Supplementary File S1. Reads were trimmed using Fastq (with poly-x trimming and no quality threshold)^66^, and aligned using Bowtie 2 (seed length 19, zero mismatches in seed)^67^. The homologs of the *asa* gene in each genome compared were detected using the DIAMOND blast search algorithm^68^. Normalized read counts (RPKM) were calculated using the BEDTools coverage tool^69^. The reads in EHEC are visualized as bam-files in Artemis 17.0 (Wellcome Sanger Institute)^70^ as the sum signal over two biological replicates.

### Test of promoter activity using pProbe-NT

A region 160 bp upstream of the start codon with a length of 92 bp was chosen for testing the activity of a putative promoter upstream of the transcriptional start site. The sequence was amplified by PCR introducing restriction enzyme cutting sites *Hind*III and *Sac*I (primer test fragment: 1Prom8220-261F*Hind*III, AGC AAG CTT GCG TGA GAT AGC CTT CGG TAT CC; 1Prom8220-173R*Sac*I, AAT GAG CTC AGC TTG TTA CAC TGC CGG CAG TC; negative control primer: 4Prom8220-493F*Hind*III, TAG AAG CTT GCG TTG GCA GGC CAG CAA TTT TGG; 4Prom8220-316R*Sac*I, CGC GAG CTC AAC GGT AAC AGC CGC TAT TTA TAC). The PCR product was cloned into pProbe-NT^33^ and transformed into *E. coli* TOP-10. A 76 bp long fragment in the terminator region of EDL933_1236 was cloned as negative control (NC I). The correctly cloned insert sequences were confirmed by Sanger sequencing (Eurofins, Ebersberg). The promoter activity tests were conducted from overnight cultures of *E. coli* TOP-10 + pProbe-NT-insert (including a second negative control, NC II, using pProbe-NT without insert). Cultures were inoculated (1:100) in 10 ml medium + 50 µg/ml kanamycin and grown at 37 °C, 150 rpm in LB, LB + 450 mM NaCl or LB + 10 mM L-arginine. Because of the slow growth rate in L-arginine, it was decided to use an L-arginine concentration half of that used in competitive growth experiments above. The cells were harvested at OD_600_ = 0.8. The pellet was washed with PBS and resuspended in 1 ml PBS. The OD_600_ was adjusted to either 0.3 or 0.6. The fluorescence was measured in black microtiter plates (four technical replicates, 200 µl volume) in Wallac Victor^3^ (Perkin Elmer Life Science, excitation 485 nm, emission 535 nm, measuring time 1 s). The background activity (*E. coli* TOP-10 without vector) was subtracted from measured values. Each condition was measured in biological triplicate.

### Determination of transcriptional start (+1) sites using 5′ RACE and Cappable Seq

The transcriptional start site of *asa* gene expression was determined with two independent methods. Cappable Seq experiments were conducted in three biological replicates following Ettwiller *et al*.^44^. The 5′ RACE was conducted in two experiments, either using EHEC or *E. coli* TOP-10. In the first experiment, EHEC were grown in LB medium. In the second experiment, *E. coli* TOP-10, transformed with either pProbe-NT or pProbe-NT plus the promoter fragment, were grown in LB + 450 mM NaCl. Both experiments were conducted as follows: an overnight culture was inoculated 1:300 in fresh medium incubated at 37 °C, 150 rpm. After OD_600_ = 0.8 was reached, 500 µl of the culture were harvested and total RNA was extracted with the SV total RNA Isolation System. The DNA was digested using 2U TURBO^TM^ DNase (Thermo Fisher Scientific). The 5′-ends were amplified from 1 µg RNA with the 5′/3′ RACE Kit, 2^nd^ Generation (Roche). The primer of the first experiment (ex1) anneal in *asa* (OLOZ1238+235R, GGA ATG GGT GAC GTA AT; OLOZ1238+207R, CAC TGG GCG GAA CGG CGT TA; OLOZ1238+179R, TCT TCT GCT GCT CTG CAT ATG TGC), those of the second experiment (ex2) anneal in GFP (RACEGFP+284R, TCC TGT ACA TAA CCT TCG G; RACEGFP+192R, AAA GTA GTG ACA AGT GTT GGC C; RACEGFP+117R, GTA TGT TGC ATC ACC TTC ACC TC). The dominant PCR products were excised from the agarose gel, purified with GenElute^TM^ Gel Extraction Kit (Sigma Aldrich) and Sanger sequenced (primers ex1: OLG8220+25R, TTT GAT CTC CTC TGG CGC TA, and ex2: RACEGFP+117R; Eurofins, Ebersberg).

### Western blot

The *asa* encoded protein Asa (expected size: 10 kDa) was detected by Western blot with a C-terminal SPA tag^34^. The vector pBAD-*myc*/His C was used as expression vector. The gene *asa* was cloned without the stop codon (primer: 8220+1F-*Nco*I; OGC243R-*Hind*III, GCC AAG CTT TCT GTC TGC CGG AAT GGG TG) to include a downstream SPA tag (primer: SPA-tag-F-*Hind*III, ATC AAG CTT ACA AGA GAA AAA AGA ATT TCA TAG CCG TCT; SPA-tag-R-*Sal*I, TTC GTC GAC CTA CTT GTC ATC GTC ATC CTT GTA GTC GAT GTC ATG; expected size of the tag is 7.5 kDa). The two SPA primers include the complete tag and were merged by PCR. The cloned sequence was checked by Sanger sequencing (primer: pBAD-C+165F; Eurofins, Ebersberg). For overexpression, an overnight culture of *E. coli* TOP-10 + pBAD-*asa*-SPA was inoculated (1:100) in LB + 120 ng/µl ampicillin and incubated to an OD_600_ = 0.3. The Asa expression was induced with 0.002% arabinose and aliquots of the culture were taken after 0.5 h, 1 h, 1.5 h, 2 h, 3 h and 4 h. The volume was adjusted to receive the same OD_600_ as at time point 0.5 h. Each aliquot was centrifuged (16,000× *g*, RT, 2 min) and the pellet was boiled in 50 µl 3× SDS sample buffer (compounded after Tricine Sample Buffer, BioRad) at 95 °C for 10 min. The Tris-Tricine-SDS PAGE was modified according to Schägger^71^. The Tris-Tricine-SDS gel consists of a 16% running gel (4.8 ml acrylamide bisacrylamide 40% (Carl Roth), 4.05 ml 3× gel buffer, 1.2 ml glycerin (85%, Merck), 1.95 ml H_2_O, 8 µl TEMED (Carl Roth), 80 µl APS (10%, Carl Roth)) and a 4% stacking gel (0.6 ml acrylamide bisacrylamide 40%, 1.5 ml 3× gel buffer, 3.9 ml H_2_O, 9 µl TEMED, 90 µl APS 10%). The SDS PAGE was conducted using cathode buffer (0.1 M Tris (Carl Roth), 0.1 M Tricine (Carl Roth), 0.1% SDS (Serva), pH 8.25) and anode buffer (0.1 M Tris, 0.0225 M HCl (Merck), pH 8.8) at 35 mA per gel for 2–3 h. The Spectra Low Range Marker (Thermo Fisher Scientific) was used to determine the protein size. The positive-control was pBAD-*gst*-SPA expressing the glutathione S-transferase (GST) of 22 kDa. For Western blot, the gel was first equilibrated in blotting buffer (0.05 M glycin, 0.4 M Tris, 1% SDS, 20% methanol) for 10 min. The PVDF membrane (Immobilon-P^SQ^, 0.22 µm, Merck) was incubated in 100% methanol for 15 s, in H_2_O for 5 min and in 1× blotting buffer for 10 min. Blotting occurred in the SemiDry Blotter Pegasus (Phase GmbH) at 12 V for 20 min. The membrane was washed with 3% TCA (Carl Roth) for 5 min and in H_2_O for 5 min. Next, the membrane was incubated overnight (4 °C, shaking) in 25 ml 1× TBS-T (10 mM Tris pH8, 0.15 M NaCl, 0.05% Tween20 (Sigma Aldrich)) with 1.25 g skim milk powder. The membrane was washed 3 times in TBS-T each for 10 min and incubated with AntiFLAG M2-AP antibody (10 ml TBS-T + 10 µl antibody) at RT for 1 h under shaking. The membrane was washed 6 times each 5 min with TBS-T and 5 min in reaction buffer (0.1 M Tris, 4 mM MgCl_2_, pH 9.5). The protein was visualized in 10 ml reaction buffer + 100 µl NBT (50 mg nitro blue tetrazolium (Applichem) in 1 ml 70% dimethyl formamide (Sigma Aldrich)) + 125 µl BCIP (20 mg 5-bromo-4-chloro-3-indolyl phosphate (Carl Roth) in 1 ml dimethyl formamide) by shaking of about 10 to 60 s. The reaction was stopped with 3% TCA after bands appeared visually.

### Bioinformatic methods

Homologous proteins were searched with blastp (NCBI, refseq database, E-value cut-off 10^-10^)^72^. Protein family domain search was used as implemented in PredictProtein^73^ using the Pfam database^74^ and conserved domains were searched in the CD database^75^.

Protein features of putative proteins encoded by *asa* were predicted using PredictProtein with the following programs: ISIS (DNA and protein binding sites)^76^, METADISORDER (disordered regions)^77^, PROFphd (secondary structure, solvent accessibility)^78^, DISULFIND (disulfide bonds)^79^, PHDhtm (transmembrane helices, accuracy >86%)^80^ and LocTree3 (cellular localization)^81^.

The sequence 300 bp upstream of the transcriptional start site was searched for a σ^70^ factor binding site with BPROM (Softberry)^82^ and manually for the consensus sequences of alternative promoters. The strength of the σ^70^ promoter was determined by BPROM as linear discriminant function, LDF. The threshold for successful promoter prediction is an LDF of 0.2. The region 600 bp downstream of the stop codon was searched for rho-independent terminators using FindTerm (Softberry)^83^.

Ribosome binding sites were identified according to Ma *et al*.^84^. The region 30 bp upstream of the start codon was checked for ribosome binding sites using a Gibbs free energy cut-off of −4.4 kcal/mol.

## Electronic supplementary material

Supplementary Files
