# Supplementary material for: The novel EHEC gene asa overlaps the TEGT transporter gene in antisense and is regulated by NaCl and growth phase
Source: Sci Rep. 2018 Dec 14;8:17875. doi: 10.1038/s41598-018-35756-y (PMC6294744; doi:10.1038/s41598-018-35756-y)
Supplement: Supplementary file 1 — Supplementary Files [file 41598_2018_35756_MOESM1_ESM.pdf]

Supplementary Files

The novel EHEC gene *asa* overlaps the TEGT transporter gene in antisense and is regulated by NaCl and growth phase

Sonja Vanderhaeghen[1], Barbara Zehentner[1], Siegfried Scherer[1,2], Klaus Neuhaus[1,3*] and Zachary Ardern[1]

[1] Lehrstuhl für Mikrobielle Ökologie, Wissenschaftszentrum Weihenstephan, Technische Universität München, Weihenstephaner Berg 3, 85354 Freising, Germany

[2] ZIEL – Institute for Food & Health, Technische Universität München, Freising, Germany

[3] Core Facility Microbiome/NGS, ZIEL – Institute for Food & Health, Technische Universität München, Weihenstephaner Berg 3, 85354 Freising, Germany

*email for correspondence: neuhaus@tum.de

Table S1: Quantification cycles (cq) of *asa* gene expression

Table S2: List of bacteria and accession numbers

File S1: Bash script for the ribosome profiling (RIBOseq)

Figure S1: Amino acid sequence of the *asa* encoded peptide

Figure S2: Expression signals of *asa* homologues in *E. coli* and *S. enterica*

Figure S3: Genomic organization of the region around *asa* and homologues in the other strains

Figure S4: Promoter regions of *asa* homologues in *E. coli* and *S. enterica*

Figure S5: Growth curves of EHEC EDL933

Figure S6: Standard curves to determine the primer efficiencies for RT-qPCR

Supplementary table S1

Quantification cycles (cq) of *asa* gene expression after growth in LB medium or LB + 450 mM NaCl determined by qPCR. The quantification of gene expression was normalized to 16S rRNA (Δcq). A negative control (neg. ctrl.) for gene expression was a 59 bp long region without any RNAseq signal. All samples were tested to be DNA-free by qPCR without reverse transcription (RT). No detection of a PCR product via fluorescence within 40 cycles is marked as 'not available' (NA). All samples were measured in each run in four technical and three biological replicates.

Time point	Medium	cq (gene)	cq (16S rRNA)	Δcq = cq (gene) – cq (16S rRNA)	mean	cq (16S rRNA) without RT
Early exponential phase	$LB_{Replicate1}$	19.0	11.4	7.6	7.67 ± 0.15	32.4
	$LB_{Replicate2}$	19.4	11.6	7.8		28.7
	$LB_{Replicate3}$	19.3	11.7	7.6		29.0
	$NaCl_{Replicate1}$	18.7	9.6	9.1	9.35 ± 0.47	35.0
	$NaCl_{Replicate2}$	17.7	8.6	9.0		38.7
	$NaCl_{Replicate3}$	17.9	8.0	9.9		35.4
Exponential phase	$LB_{Replicate1}$	19.5	14.2	5.3	5.67 ± 0.72	NA
	$LB_{Replicate2}$	18.3	13.1	5.2		38.2
	$LB_{Replicate3}$	19.5	13.0	6.5		NA
	$NaCl_{Replicate1}$	18.2	14.5	3.6	3.53 ± 0.85	39.9
	$NaCl_{Replicate2}$	18.9	16.2	2.7		NA
	$NaCl_{Replicate3}$	17.2	12.9	4.3		38.6
	$LB_{neg. ctrl.}$	32.0	15.6	16.4	-	16.6

Supplementary table S2

List of bacteria, genome accession numbers (GenBank, NCBI), and RNAseq and RIBOseq data accession numbers (SRA, NCBI) used for transcriptional and translational analyses of *asa*.

Organism	GenBank accession number	SRA accession number		Publication
E. coli O157:H7 strain EDL933	CP008957.1	RNAseq	Replicate 1: SRR5266617<br>Replicate 2: SRR5266619	Landstorfer, 2014[54]
		RIBOseq	Replicate 1: SRR5266618<br>Replicate 2: SRR5266620	
E. coli O157:H7 strain Sakai	NC_002695	RNAseq	Replicate 1: SRR5874481<br>Replicate 2: missing	Hücker et al. 2017[3]
		RIBOseq	Replicate 1: SRR5874484<br>Replicate 2: missing	
E. coli LF82	NC_011993.1	RNAseq	N/A	unpublished
		RIBOseq	N/A	
E. coli K12 substrain MG1655	NC_000913.3	RNAseq	Replicate 1: SRR4023277<br>Replicate 2: SRR4023278<br>Replicate 3: SRR4023279	Hwang and Buskirk 2017[55]
		RIBOseq	Replicate 1: SRR4023274<br>Replicate 2: SRR4023275<br>Replicate 3: SRR4023276	
E. coli K12 substrain MC4100	NZ_HG738867.1	RNAseq	Replicate 1: SRR2016456<br>Replicate 2: SRR2016464	Bartholomäus et al. 2016[56]
		RIBOseq	Replicate 1: SRR2016457<br>Replicate 2: SRR2016465	
S. enterica 14028S	NC_016856.1	RNAseq	SRR4417739	Baek et al. 2017[8]
		RIBOseq	SRR4417735	

```
# Supplementary file S1
# Bash script for the ribosome profiling (RIBOseq) analyses, with input
file instructions and tool version numbers.


#!/bin/bash

# CALCULATE RPKM AND RCV FOR HOMOLOG OF GENE OF INTEREST

#
### TO RUN:
#
# bash RiboSeq_and_RPKMs.bash datasets.txt ;


#
### REQUIRED FILES:

# dataset file, e.g. datasets.txt:
# sample line from dataset input file, below  - 4 tab-separated columns
(no header);   Condition   RNASeq_file   RiboSeq_file    Genome_file
# LB      SRR2016456.fastq        SRR2016457.fastq
GCF_000499485.1_MYMC4100_genomic.fna


#'OLG file' (set below) is a fasta file with the amino acid sequence of
the protein of interest
# genomic.fna and feature_table.txt files are downloaded from NCBI's FTP
site


#
### TOOLS USED:

#     bedtools v2.27.1-2-ge5ad7e4
#     fastp version 0.12.6
#     bowtie2 version 2.2.6
#     emboss version EMBOSS:6.6.0.0
#     diamond version 0.9.14


dataset="$1" ;
adapter="-" ;
OLG_file=8220-7900.fasta ;
cores=4 ;

###

#FUNCTIONS:

linear () { awk '!/^>/ { printf "%s", $0; n = "\n" } /^>/ { print n $0; n
= "" } END { printf "%s", n }'; } ;

####################################################


cat $dataset |
while read -r sample rnaseq riboseq genome ;

do
```

```
ft=${genome%_*}_feature_table.txt ;
chromosome=$(cat $genome | head -1 | awk '{print $1}' | sed -e "s|>||g")
;

for input in $rnaseq $riboseq ;


do
echo $genome $input $adapter ;


echo "unzip "$input".gz" ;
test ! -e "$input" && unpigz -p 4 "$input".gz ;



# Finding tRNA & rRNA regions of genome
test ! -e $chromosome.fna &&  echo "find tRNA and rRNA regions in genome
to exclude later" ;

test ! -e $chromosome.fna && faidx -x $genome ;

#BED file of tRNA and rRNA positions, from feature table
test ! -e $chromosome-excluded-RNAs.bed && cat $ft | awk -F "\t"  '{if
($1=="gene" && $7=="'$chromosome'") print}' |
awk -F "\t" '{if ( $2=="rRNA" || $2=="tRNA") print $7 "\t" ($8-1) "\t" $9
"\t" $17 "\t" "0" "\t" $10 }' > $chromosome-excluded-RNAs.bed ;



#############################################################

# PRE-PROCESSING OF FASTQ FILE
# removing adapter sequences

test $adapter != "-" && test ! -e ${input%.*}-fastp.fastq && fastp -i
$input -x -Q -a $adapter -o ${input%.*}-fastp.fastq ;
test $adapter = "-" && test ! -e ${input%.*}-fastp.fastq && fastp -i
$input -x -Q -o ${input%.*}-fastp.fastq ;


##################################

# ALIGNING


test ! -e "$chromosome".1.bt2 && bowtie2-build "$chromosome".fna
"$chromosome" 2> /dev/null ;

test ! -e ${input%.*}.sorted.bam && bowtie2 -p $cores --local -x
$chromosome -N 0 -L 19 -U ${input%.*}-fastp.fastq |
samtools view -bh - | samtools sort - > ${input%.*}.sorted.bam ;
samtools index ${input%.*}.sorted.bam  ;

# zip fastq file and remove fastp.fastq file to save space
pigz -p $cores $input ;
rm ${input%.*}-fastp.fastq ;
```

```
################
# Remove rRNA & tRNA regions from BAM file, to create input SAM for
REPARATION
test ! -e ${input%.*}_RNAfree.bam && bedtools intersect -abam
${input%.*}.sorted.bam  -b $chromosome-excluded-RNAs.bed -v |
samtools view -hb > ${input%.*}_RNAfree_headered.bam ;



#########################################################################
###

# FIND OLG HOMOLOG

#######################


getorf -filter -sequence "$chromosome".fna -table 11 -minsize 93 -find 1
-circular Y | linear > ${genome}_ORFs.fasta ;

~/diamond/diamond makedb --in ${genome}_ORFs.fasta  -d ${genome}_ORFs  ;

~/diamond/diamond blastp -q $OLG_file -d ${genome}_ORFs -p $cores -o
tmp.txt \
-k 10000 -e 0.001 -b 1.6 -t ~/tmp -f 6 pident sseqid qseqid evalue sstart
send > /dev/null ;

hit=$(cat tmp.txt | awk '{print $2}');

cat ${genome}_ORFs.fasta | grep -A1 $hit | head -1 | awk '{print $1 "\t"
$2 "\t" $4}' | sed -e "s|[][]||g" | sed -e "s|>||g" |
awk '{if ($2<$3) print $1 "\t" $2 "\t" $3 "\t" "+" ; else print $1 "\t"
$3 "\t" $2 "\t" "-"}' |
awk '{split($1,a,"_"); print a[1] "_" a[2] "\t" $2 "\t" $3 "\t" "OLG"
"\t" "0" "\t"  $4}' > OLG.bed ;


############ Find RPKM & RCV for OLG homolog


echo $input - calculating RPKM ;

# Calculate RPKMs

reads=$(samtools flagstat ${input%.*}_RNAfree_headered.bam | grep mapped
| grep -v mate | awk '{print $1}' ) ;
millions=$(echo "$reads / 1000000" | bc -l) ;

cat $genome.fai | awk '{print $1 "\t" $2}' > genome.txt ;

# note - "+1" added to first column to convert 0-based numbering of bed
file back to 1-based numbering!
bedtools coverage -sorted -s -a OLG.bed -b
${input%.*}_RNAfree_headered.bam -g genome.txt |
awk '{if ($7>0) print ($2+1) "\t" $3 "\t" $4 "\t" $7 "\t"
$7/(($9/1000)*"'$millions'") "\t" $NF;
else print ($2+1) "\t" $3 "\t" $4 "\t" $7 "\t" "0" "\t" $NF}' >
${input%.*}_coverage.txt ;

done ;
```

```
# Calculate RCVs [final column of output]

paste ${rnaseq%.*}_coverage.txt ${riboseq%.*}_coverage.txt | awk '{if
($5>0) print $0 "\t" $11/$5 ; else print $0 "\t" "zero-RNA"}' > $sample-
RCVs.txt ;


done ;
```

Supplementary figure S1

***Ec* EDL933**	MMLLVSNKIAPEEIKMKTALIARCRAGSCRKILATIPISTTTIPAISIPPRNDISLRVVSTYAEQQKNTNAVPPSAIAITSPIPADR*
Ec Sakai	MMLLVSNKIAPEEIKMKTALIARCRAGSCRKILATIPISTTTIPAISIPPRNDISLRVVSTYAEQQKNTNAVPPSAIAITSPIPADR*
Ec LF82	MMLLVSNKIAPEEI**R**MKTALIARCRAGSCRKILATIPISTTTIPAISIPPRNDISLRVVSTYAEQQKNTNAVPPSAIAITSPIPADR*
Ec MG1655	MMLLVSNKIAPEEI**R**MKTALIARCRAGSCRKI**F**ATIPISTTTIPAISIPPRNDISLRVVSTYAEQQKNTNAVPPSAIAITSPIPADR*
Ec MC4100	MMLLVSNKIAPEEI**R**MKTALIARCRAGSCRKI**F**ATIPISTTTIPAISIPPRNDISLRVVSTYAEQQKNTNAVPPSAIAITSPIPADR*
Se 14028S	MMLLVS**YR**IAPEEI**RIN**TALIARC**N**AGSCRKI**F**ATIPISTTTIPAI**N**IPPR**K**DISLRVV**R**T*-------------------------

Amino acid sequence of the *asa* encoded peptide in strain EDL933 (*Ec* EDL933) and homologous peptides of the *E. coli* strains O157:H7 Sakai (*Ec* Sakai), LF82 (*Ec* LF82), K12 substrain MG1655 (*Ec* MG1655), K12 substrain MC4100 (*Ec* MC4100) and of S*almonella enterica* 14028S (*Se* 14028S). The first amino acid is marked in green and the end using a red asterisk. Those amino acids that are changed in comparison to the original *asa* encoded peptide are marked in blue letters.

Supplementary figure S2

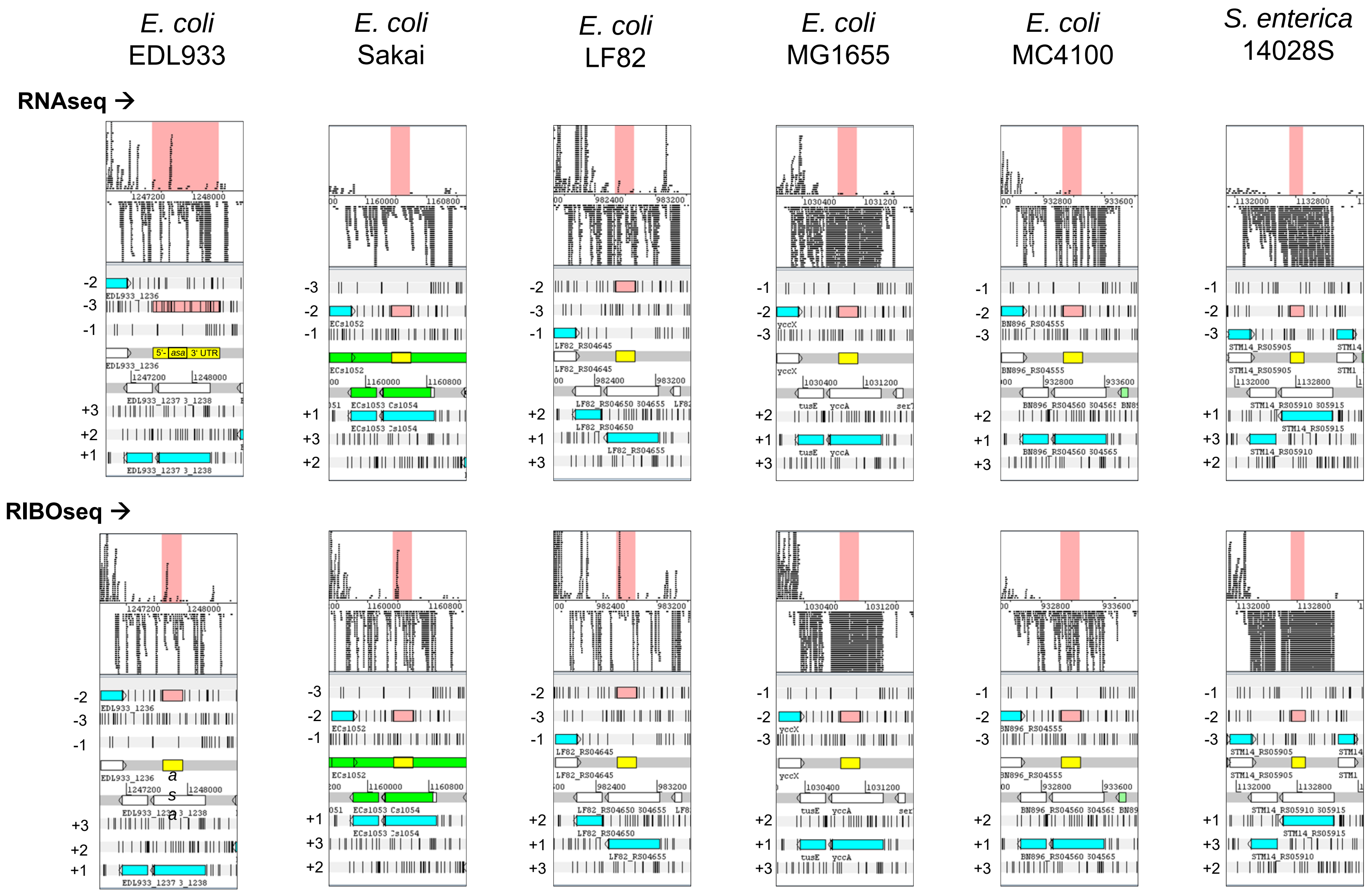

Expression signals of *asa* homologues in *E. coli* and *S. enterica*. RNAseq and ribosomal footprinting (RIBOseq) signals of *asa* homologues were obtained from our lab (*E. coli* O157:H7 Sakai and *E. coli* LF82) or downloaded from SRA database (*E. coli* K12 substrain MG1655, *E. coli* K12 substrain MC4100 and *S. enterica* 14028S). The reads are visualized as sum signal of two biological replicates using bam files in Artemis Release 17.0[61]. The putative overlapping homologue of *asa* is highlighted in salmon or yellow.

Supplementary figure S3

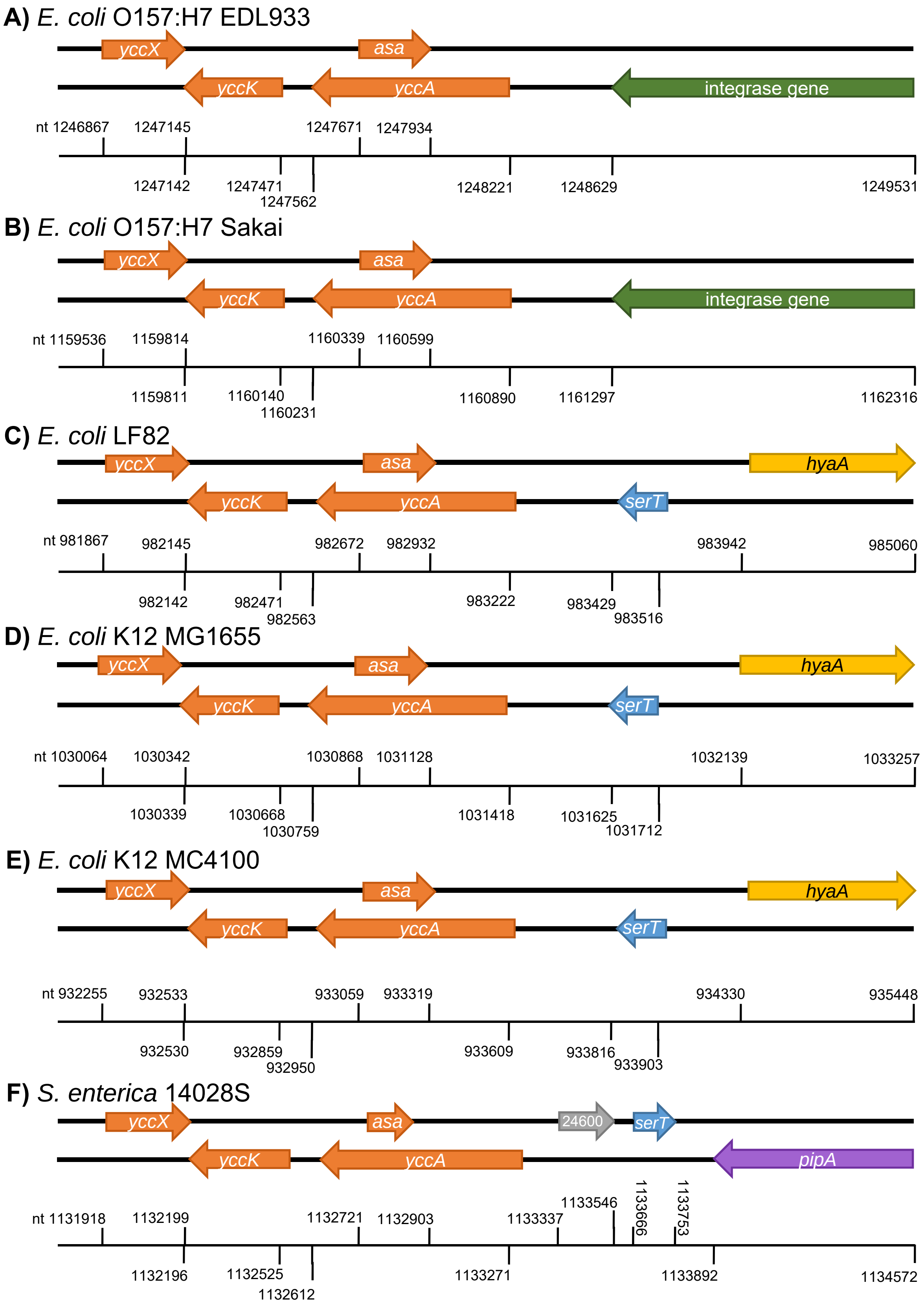

Genomic organization of the region around *asa* and homologues in bacteria different from EDL933. Coordinates below each panel are given in respect to each genome. (**A**) *E. coli* O157:H7 strain EDL933, (**B**) *E. coli* O157:H7 strain Sakai; (**C**) *E. coli* LF82; (**D**) *E. coli* K12 substrain MG1655; (**E**) *E. coli* K12 substrain MC4100; (**F**) *S. enterica* 14028S. Gene products in orange: *yccX* = acylphosphatase, *yccK* = sulfurtransferase TusE, *yccA* = TEGT family transporter; in green: phage integrase; in blue: *serT* = Serine tRNA; in yellow: *hyaA* = hydrogenase-1 small chain; in grey: 24600 = hypothetical protein; in violet: *pipA* = PipA/GogA/GtgA family type III secretion system effector. Same gene names encode the same product, but are not necessarily the names used in the respective genome of the organism.

Supplementary figure S4

Promoter regions of *asa* homologues in *E. coli* and *S. enterica.* Shown are *E. coli* O157:H7 strain EDL933 (*Ec* EDL933) compared to four *E. coli* strains (*Ec* Sakai, *Ec* LF82, *Ec* MG1655 and *Ec* MC4100) and to *S. enterica* (*Se* 14028S).
There are three +1 sites (green arrows) and three putative promoters (σ^{70}: yellow, σ^{38}: blue) in EDL933. Nucleotides that do not fit to the consensus sequence TATAAT/TTGAAT (σ^{70}) or CTACACT (σ^{38}) are written in small letters.

σ^{70} promoter

+1 +1 +1

***Ec* EDL933**	CCGTTtctaTCTCTTTACCTTCGAAGATCAGcATAATT-ATTACTACCTTAATCAGACT
Ec Sakai	CCGTTtctaTCTCTTTACCTTCGAAGATCAGcATAATT-ATTACTACCTTAATCAGACT
Ec LF82	CCGTTtctaTCTCTTTACCTTCGAAGATCAGcATAATTCTTTACTACCTTAATCAGACT
Ec MG1655	CCGTTtctaTCTCTTTACCTTCGAAGATCAGcATAATT-ATTACTACCTTAATCAGACT
Ec MC4100	CCGTTtctaTCTCTTTACCTTCGAAGATCAGcATAATT-ATTACTACCTTAATCAGACT
Se 14028S	CTATcagtaCTGATTTCTTTACCTTCAAAGATcaAcaT-AACGTTTCACTGCGTAATCA

σ^{38} promoter

+1 +1 +1

***Ec* EDL933**	CCGTTTCTATCTCTTTACCTTCGAAGATCAGCaTAatt-aTTACTACCTTAATCAGACT
Ec Sakai	CCGTTTCTATCTCTTTACCTTCGAAGATCAGCaTAatt-aTTACTACCTTAATCAGACT
Ec LF82	CCGTTTCTATCTCTTTACCTTCGAAGATCAGCaTAattcTTTACTACCTTAATCAGACT
Ec MG1655	CCGTTTCTATCTCTTTACCTTCGAAGATCAGCaTAatt-aTTACTACCTTAATCAGACT
Ec MC4100	CCGTTTCTATCTCTTTACCTTCGAAGATCAGCaTAatt-aTTACTACCTTAATCAGACT
Se 14028S	CTATCAGTACTGATTTCTTTACCTTCAAAGATCTACAt-aACGTTTCACTGCGTAATCA

σ^{38} promoter

+1 +1 +1

***Ec* EDL933**	CCGTTTCTATCTCTTTACCTTCGAAGATCAGCATAATT-ATTACTACctTAATCAGACT
Ec Sakai	CCGTTTCTATCTCTTTACCTTCGAAGATCAGCATAATT-ATTACTACctTAATCAGACT
Ec LF82	CCGTTTCTATCTCTTTACCTTCGAAGATCAGCATAATTCTTTACTACctTAATCAGACT
Ec MG1655	CCGTTTCTATCTCTTTACCTTCGAAGATCAGCATAATT-ATTACTACctTAATCAGACT
Ec MC4100	CCGTTTCTATCTCTTTACCTTCGAAGATCAGCATAATT-ATTACTACctTAATCAGACT
Se 14028S	CTATCAGTACTGATTTCTTTACCTTCAAAGATCAACAT-AACGtTtCACTGCGTAATCA

Supplementary figure S5

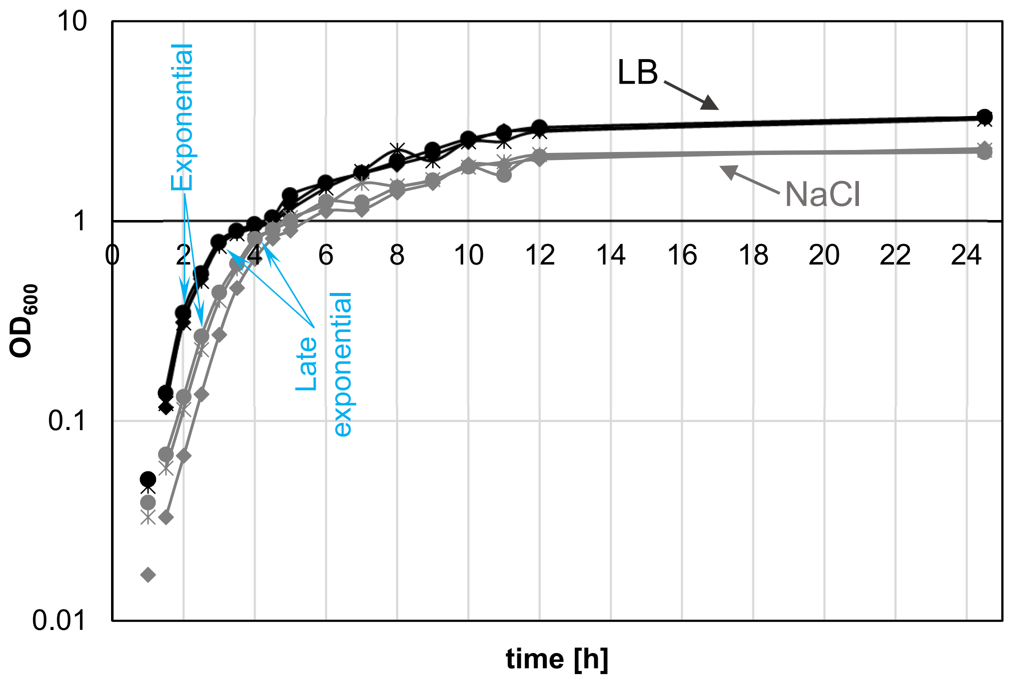

Growth curves of EHEC EDL933 grown in LB (black) and in LB + 450 mM NaCl (grey). Shown are three replicates (●, ◆, ✳). Aliquots (~10^8 cells) were taken at exponential phase (OD_{600} = 0.2 - 0.3) and at late exponential phase (OD_{600} = 0.7 - 0.8) for RT-qPCR as indicated by the blue arrows.

Supplementary figure S6

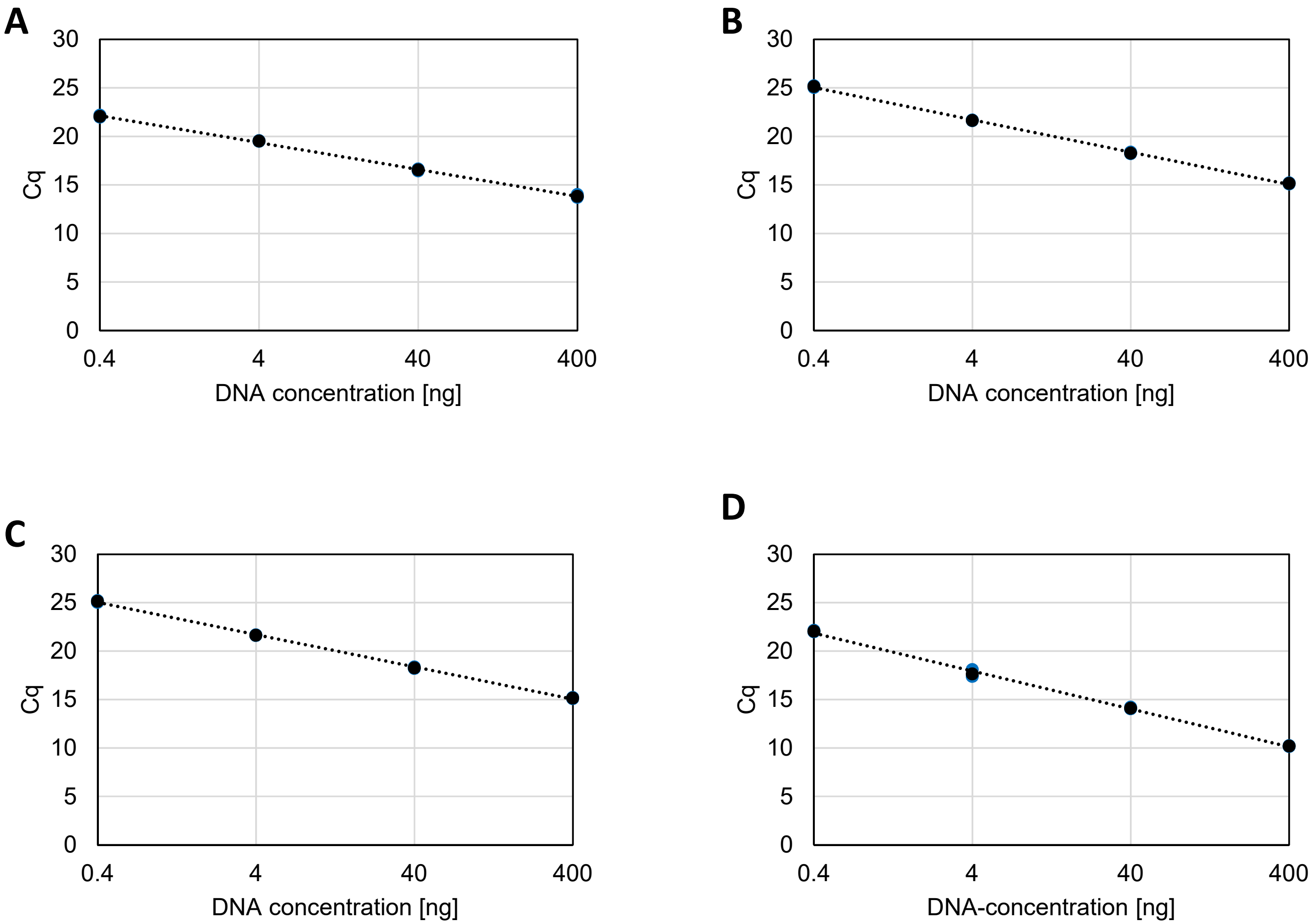

Standard curves to determine the primer efficiencies for RT-qPCR. The average quantification cycle (cq) of three technical replicates (black) was plotted against the following genomic DNA amounts: 0.4 ng, 4 ng, 40 ng and 400 ng. The individual data points of each replicate is shown in blue. (**A**) *asa*, annealing temperature 61°C, primer qPCR-OLG8220+25F / qPCR-OLG8220+191R; (**B**) negative control, annealing temperature 58°C, primer qPCR-neg8220F/qPCR-neg8220R; (**C, D**) 16S rRNA gene used for normalizing, annealing temperatures 61°C or 58°C respectively, primer rrHF/rrHR.
